# BoLA-DRB3 gene haplotypes show divergence in native Sudanese cattle from taurine and indicine breeds

**DOI:** 10.1038/s41598-021-96330-7

**Published:** 2021-08-25

**Authors:** Bashir Salim, Shin-nosuke Takeshima, Ryo Nakao, Mohamed A. M. Moustafa, Mohamed-Khair A. Ahmed, Sumaya Kambal, Joram M. Mwacharo, Abeer M. Alkhaibari, Guillermo Giovambattista

**Affiliations:** 1 Department of Parasitology, Faculty of Veterinary Medicine, University of Khartoum, Khartoum-North, Sudan; 2 Department of Food and Nutrition, Faculty of Human Life, Jumonji University, 2-1-28 Sugasawa, Niiza, Saitama Japan; 3 Laboratory of Parasitology, Department of Disease Control, Graduate School of Veterinary Medicine, Hokkaido University, Sapporo, Japan; 4 Department of Genetics and Animal Breeding, Faculty of Animal Production, University of Khartoum, Khartoum, Sudan; 5 National University, Biomedical Research Institute, Khartoum, Sudan; 6 International Centre for Agricultural Research in the Dry Areas (ICARDA), Beirut, Lebanon; 7 Department of Biology, Faculty of Science, University of Tabuk, Tabuk, 71491 Kingdom of Saudi Arabia; 8 Facultad de Ciencias Veterinarias UNLP, IGEVET - Instituto de Genética Veterinaria (UNLP‐CONICET LA PLATA), La Plata, Argentina

**Keywords:** Evolution, Genetics, Immunology, Molecular biology

## Abstract

Autochthonous Sudanese cattle breeds, namely Baggara for beef and Butana and Kenana for dairy, are characterized by their adaptive characteristics and high performance in hot and dry agro-ecosystems. They are thus used largely by nomadic and semi-nomadic pastoralists. We analyzed the diversity and genetic structure of the BoLA-DRB3 gene, a genetic locus linked to the immune response, for the indigenous cattle of Sudan and in the context of the global cattle repository. Blood samples (n = 225) were taken from three indigenous breeds (Baggara; n = 113, Butana; n = 60 and Kenana; n = 52) distributed across six regions of Sudan. Nucleotide sequences were genotyped using the sequence-based typing method. We describe 53 alleles, including seven novel alleles. Principal component analysis (PCA) of the protein pockets implicated in the antigen-binding function of the MHC complex revealed that pockets 4 and 9 (respectively) differentiate Kenana-Baggara and Kenana-Butana breeds from other breeds. Venn analysis of Sudanese, Southeast Asian, European and American cattle breeds with 115 alleles showed 14 were unique to Sudanese breeds. Gene frequency distributions of Baggara cattle showed an even distribution suggesting balancing selection, while the selection index (ω) revealed the presence of diversifying selection in several amino acid sites along the *BoLA-DRB3* exon 2 of these native breeds. The results of several PCA were in agreement with clustering patterns observed on the neighbor joining (NJ) trees. These results provide insight into their high survival rate for different tropical diseases and their reproductive capacity in Sudan's harsh environment.

## Introduction

There is a consensus among population geneticists that the Sudanese cattle populations belong to the humped Zebu cattle breed and are classified into two principal varieties: northern Sudan and Nilotic^1,2^. The Kenana and Butana breeds are the best-known milk-producing northern Sudan Zebu breeds^3–5^ with milk yield of over 1500 kg per lactation^6–8^.

The Kenana breed, predominantly found in the Blue Nile state, is distinguished by a light blue-gray coat color with darker hooves and head. The Butana breed of the Batahin and Shukria tribes inhabits the desert area between the Blue Nile and the River Atbara, and has a red-coat^1^. A third breed, the Baggara, is raised by Baggara Bedouin pastoralists. It is the major fattening Zebu cattle breed of northern Sudan, found mostly in west Sudan (Darfur and Kordofan regions), Niger, Chad, Cameroon and Nigeria. They have short horns and a large hump, with a red or dark red coat in Daeinawi Aka Messairi/Rezaigi population or white markings or black markings in the Nyalawi population^9^.

The immune system in vertebrates evolved to defend against invasive pathogens^10^ and thus it is not surprising that genetic factors are implicated in disease susceptibility in cattle. The major histocompatibility complex (MHC) is a major component of the adaptive immune system, with MHC genes encoding the cell-surface glycoproteins that bind small peptide fragments derived from host- and pathogen-expressed proteins via proteolysis^11^. Animal breeders are becoming more interested in the MHC due to its association with genetic resistance and susceptibility to a wide variety of diseases^12^. Genetic characterization of MHC polymorphism can help reduce the occurrence and severity of infectious diseases in domestic animal and cattle breeding programs^13^. The association of MHC with diseases in ruminants is well documented^14–19^. The MHC genes are assigned to *Bos taurus* autosome chromosome 23 (BTA 23)^14,15^, and is known as the Bovine Leukocyte Antigen (BoLA). Recently Kim et al.^16^, investigated five African breeds for the identification of common and unique African genome-specific selection signatures and compared them with commercial breeds. They identified six BoLA haplotype blocks, and that the major African cattle haplotypes correspond to minor haplotypes in commercial cattle. The BoLA molecules' extensive structural polymorphism is responsible for the large differences in cattle’s immune response to infectious agents. For example, *BoLA-DRB3* polymorphisms had been associated with tick infestation resistance^16^. MHC research may also assist in the formation and design of synthetic peptide-based vaccines containing one or more pathogen T-cell epitope.

Polymerase chain reaction-sequence based typing (PCR-SBT) to assess the genetic diversity of the *BoLA-DRB3* gene has been done with only a few breeds and cross-breeds from Europe, Asia and the Americas^17–30^. Target next generation sequencing (Target-NGS)^31^, the most powerful tools used to identify diversity of *BoLA-DRB3* alleles in cattle breeds, has also not been extensively applied. This is despite the central role of the *BoLA-DRB3* alleles in the immune response of cattle. Until now, private African *BoLA-DRB3* alleles have been reported by authors using indirect techniques, such as polymerase chain reaction follow by restriction fragment length polymorphism (PCR–RFLP), followed by cloning and sequencing^32–34^. These studies focused mainly on screening and analysis of only a few animals from a small selection of African breeds (e.g. Sanga, Kenana, Butana).

Previous work showed the presence of a high number of private alleles in native breeds. Consequently, there are still a number of breeds that remain uncharacterized, and this number only increases when local native bovine breeds are considered^19,21–23^.

Here we examine patterns of genetic variation of BoLA-DRB3 alleles in Baggara, Butana and Kenana native cattle breeds of Sudan and compare these with commercial breeds to both identify any unique alleles in Sudanese native cattle breeds and to provide information on these alleles to enable further studies of disease susceptibility and resistance, particularly for designing improved breeding schemes.

## Results

### Distribution of *BoLA-DRB3* alleles in selected native Sudanese cattle breeds

**Figure 1:**
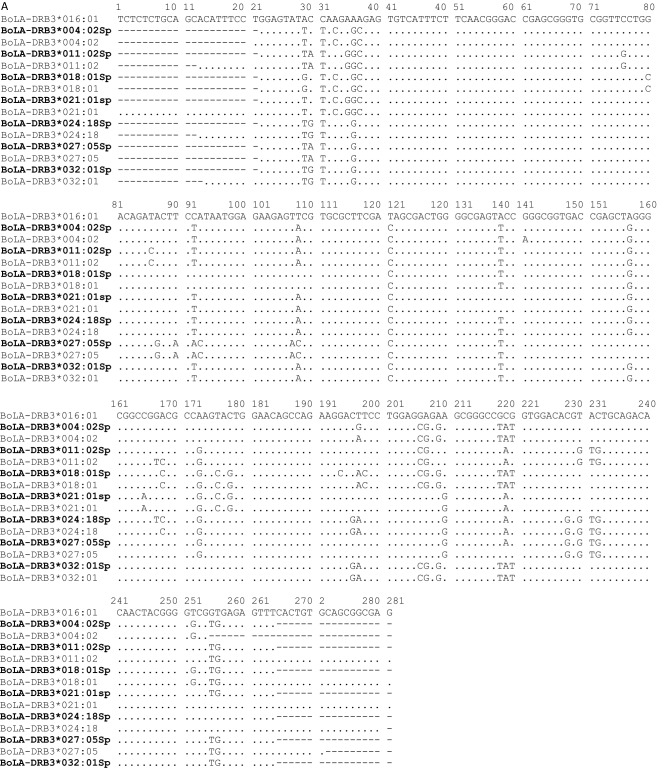

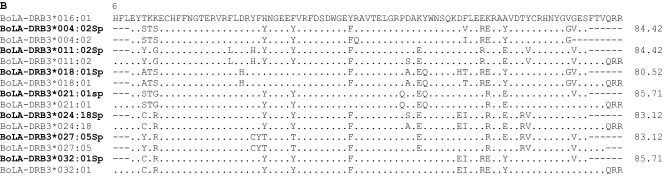
Alignment of the nucleotide (**A**) and the predicted amino acid (**B**) sequences of the β1 domain encoded by seven new *BoLA-DRB3* alleles (accession numbers *,* LC569725 for BoLA-DRB3*004:02Sp, LC569726 for BoLA-DRB3*011:02Sp, LC569729 for BoLA-DRB3*018:01Sp, LC569731 for BoLA-DRB3*021:01sp, LC569733 for BoLA-DRB3*024:18Sp, LC569735 for BoLA-DRB3*027:05sp, and LC569739 for BoLA-DRB3*032:01sp) derived from 225 Sudan native cattle (113 animals of the Baggara native, 60 Butana, and 52 Kenana Sudan native cattle breeds). New alleles are indicated in bold. Numbering refers to amino acid positions in the mature protein. Nucleotide and amino acid residues identical to those encoded by the *BoLA-DRB3* cDNA clone NR-1 are indicated by dots (Aida et al., 1995). Missing data are indicated by dashes. Closer *BoLA-DRB3* alleles with new variants are also included in the figure. Id. = Nucleotide or amino acid identity in %.

PCR-SBT genotyping allowed us to identify 53 *BoLA-DRB3* alleles (46 previously reported variants and seven new alleles; Table 1) from the native breeds selected in this study. The number of alleles (n_a_) was 46 in Baggara cattle (40 previously reported and six new), 33 in Kenana cattle (28 previously reported and five new), and 33 in Butana cattle (28 previously reported and five new) (Tables 1 and 2). The new *BoLA-DRB3* variants were confirmed by the presence of at least three carrier animals and in two breeds, and were submitted to the DNA Data Bank of Japan (http://www.ddbj.nig.ac.jp) under accession numbers LC569724-LC569739. Nucleotide and predicted amino acid sequences of the seven new allele variants are shown in Fig. 1 and compared with the most similar *BoLA-DRB3* reported so far. All seven new *BoLA-DRB3* allele variants shared about 89.7–92.6% and 80.52–85.71% nucleotide and amino acid similarity with the *BoLA-DRB3* cDNA clone NR1, respectively (Aida, 1995).

**Table 1:** BoLA-DRB3 allele frequencies (in percentage) in native Sudanese cattle breeds.

DRB3	Baggara	Kenana	Butana
	(N = 113)	(N = 52)	(N = 60)
BoLA-DRB3*003:01	0.00	0.96	0.00
BoLA-DRB3*003:02:01	4.87	**7.69**	**5.83**
BoLA-DRB3*004:01	2.65	0.00	0.00
BoLA-DRB3*005:02	4.87	2.88	2.50
BoLA-DRB3*007:01	2.65	0.96	0.00
BoLA-DRB3*008:01	1.33	0.00	0.83
BoLA-DRB3*009:01	0.88	0.00	0.00
BoLA-DRB3*009:02	2.65	0.96	4.17
BoLA-DRB3*010:01	0.44	0.96	4.17
BoLA-DRB3*010:02	1.33	1.92	1.67
BoLA-DRB3*011:01	4.42	0.00	0.00
BoLA-DRB3*011:02	0.44	0.00	0.00
BoLA-DRB3*012:01	2.65	3.85	0.00
BoLA-DRB3*013:01	3.54	0.00	1.67
BoLA-DRB3*014:01:01	4.42	2.88	9.17
BoLA-DRB3*015:01	4.42	1.92	0.83
BoLA-DRB3*016:01	5.75	0.00	0.83
BoLA-DRB3*018:01	2.21	4.81	4.17
BoLA-DRB3*019:01	0.00	0.00	0.83
BoLA-DRB3*020:01:01	1.32	4.81	0.83
BoLA-DRB3*020:02	0.88	0.00	0.00
BoLA-DRB3*020:03	0.44	0.00	0.83
BoLA-DRB3*021:01	3.10	**7.69**	**10.83**
BoLA-DRB3*022:01	**7.08**	**6.73**	**9.17**
BoLA-DRB3*022:10	0.00	0.00	0.83
BoLA-DRB3*022:12	0.44	0.00	0.00
BoLA-DRB3*023:01	1.77	2.88	2.50
BoLA-DRB3*024:01	4.87	**14.42**	**5.00**
BoLA-DRB3*024:06	0.00	1.92	0.83
BoLA-DRB3*025:01:01	0.44	0.96	0.00
BoLA-DRB3*026:01	2.65	0.96	3.33
BoLA-DRB3*027:03	0.44	0.96	0.00
BoLA-DRB3*027:04	1.33	1.92	1.67
BoLA-DRB3*027:05	0.00	0.96	0.83
BoLA-DRB3*028:01	3.98	**7.69**	3.33
BoLA-DRB3*028:02	3.10	0.96	4.17
BoLA-DRB3*029:02	0.44	0.00	0.00
BoLA-DRB3*030:01	0.44	0.00	0.00
BoLA-DRB3*032:03	0.00	0.96	0.00
BoLA-DRB3*033:01	0.44	1.92	0.83
BoLA-DRB3*036:01	1.33	0.00	0.00
BoLA-DRB3*039:01	0.44	0.96	1.67
BoLA-DRB3*044:01	1.77	0.00	1.67
BoLA-DRB3*048:02	4.42	1.92	0.00
BoLA-DRB3*100:01	0.44	0.00	0.00
BoLA-DRB3*107:01	0.44	0.00	0.00
BoLA-DRB3*004:02sp2	0.44	0.00	**5.00**
BoLA-DRB3*011:02Sp	3.10	2.88	**5.83**
BoLA-DRB3*018:01Sp	0.44	1.92	0.00
***BoLA-DRB3021:01sp***	0.88	0.00	1.67
BoLA-DRB3*024:18Sp	0.00	3.85	0.83
BoLA-DRB3*027:05Sp	2.21	1.92	1.66
BoLA-DRB3*032:01Sp	1.33	0.96	0.00

N. number of animals analyzed; Frequent alleles in each breed are indicated in bold (> 5%); Novel alleles identified in this study are indicated in bold and underlined.

**Table 2:** Number of alleles (n_a_) and new alleles, observed (h_o_) and expected (h_e_) heterozygosity, Hardy Weinberg equilibrium (HWE) measured through F_IS_ and Slatkin's exact test in the cattle breeds studied. N = sample size.

Breed	N	n_a_	New alleles	h_o_	h_e_	HWE	Slatkin's *p* value
						F_IS_—*p* value	
Baggara	113	46	6	0.938	0.969	0.032–0.277	**0.016**
Butana	52	33	5	0.961	0.951	0.005–0.100	0.255
Kenana	60	33	5	0.950	0.954	−0.011–0.628	0.138

**Figure 2:**
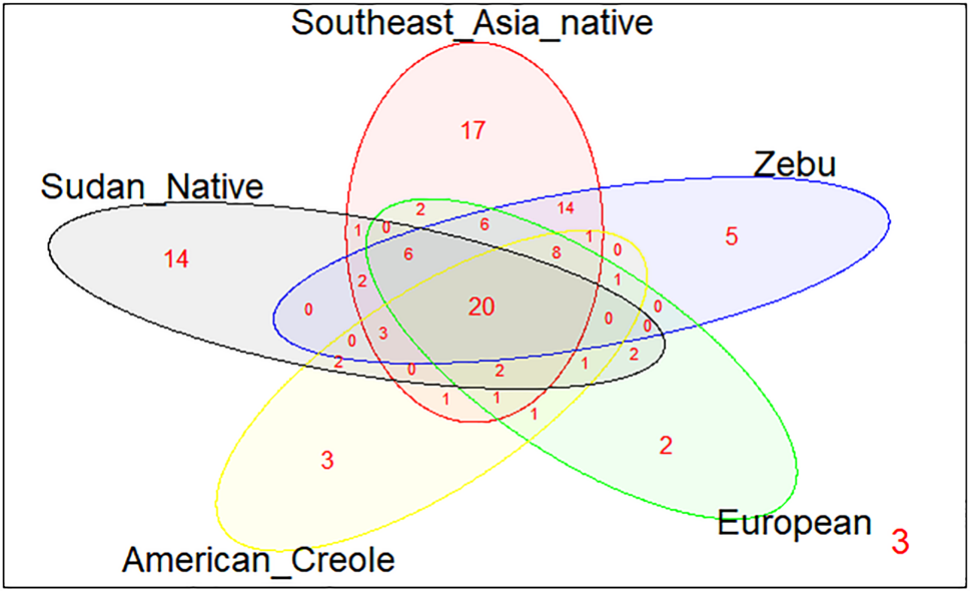
Venn plot of *BoLA-DRB3* alleles shared by Sudan native (Baggara, Kenana, and Butana); Southeast Asia (Myanmar and Philippine native breeds); indicine (Nellore, Gir, Brahman, and crossbreeds); European (Hereford, Black and Red Angus, Jersey, Shorthorn, Holstein, overo negro, overo colorado, and crossbreeds); and American Creole (Yacumeño and Hartón del Valle) cattle breeds.

**Figure 3:**
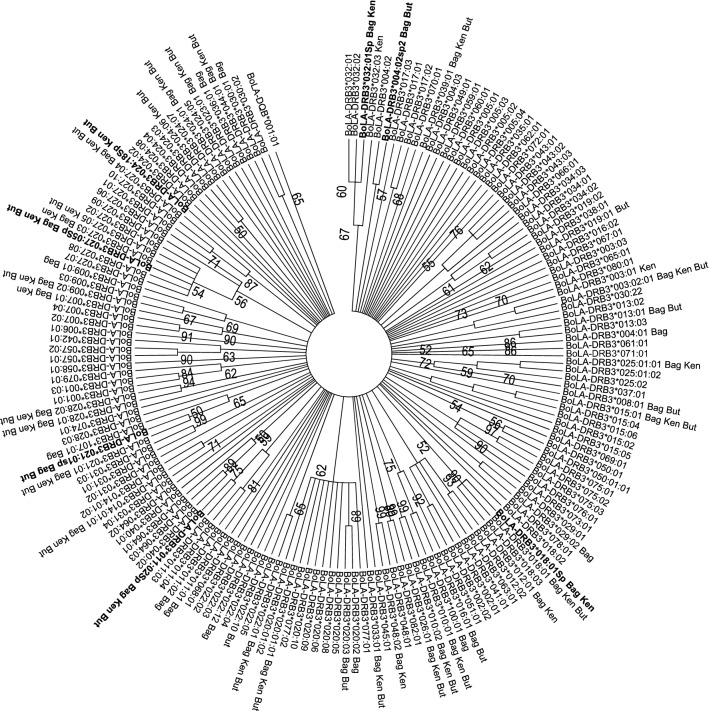
Neighbor-joining (NJ) tree constructed from the 270 bp nucleotide sequence that includes the β1 domain encoded by all reported *BoLA-DRB3* alleles and the seven new ones (BoLA-DRB3*004:02Sp2, BoLA-DRB3*011:02Sp, BoLA-DRB3*018:01Sp, BoLA-DRB3*021:01sp, BoLA-DRB3*024:18Sp, BoLA-DRB3*027:05sp, and BoLA-DRB3*032:01sp). Numbers are bootstrap percentages that support each node. Bootstrapping was carried up with 1000 replicates to access the reliability of individual branches. Bag = Baggara, But = Butana, Ken = Kenana. Arrows indicate novel alleles.

A Venn diagram was constructed using data obtained in this study and from previous reports^18,19,21,27,29^. Data were grouped in terms of the breed’s geographical origin as follows: native Sudanese; Southeast Asian; Zebu; European; and American Creole cattle breeds (Fig. 2). This analysis revealed that out of the 115 alleles identified in the five cattle groups, fourteen were unique to native Sudanese breeds (Fig. 2), four of which exhibited gene frequencies that were higher than 0.5%, representing about 26% of the 53 alleles detected in the native Sudanese cattle. In addition, two other variants were only present in native Sudanese and American Creole breeds, while six other alleles were only found in Sudanese cattle populations and American Creole or Southeast Asian native or Zebu breeds, or a combination of these groups. In addition, the *BoLA-DRB3* NJ tree, including all the previously reported alleles and the seven new variants, showed that the variants detected in Sudanese cattle populations were interspersed among the various clusters (Fig. 3). A similar result was observed when the *BoLA-DRB3* tree was inferred using amino-acid residues located in the antigen-binding site (ABS) (Fig. S1).

As shown in Fig. S2, the native Sudanese cattle breeds have an even gene frequency distribution, with a high number of alleles with low frequency. Low allele frequency was particularly noticeable in the Baggara breed. Only two, five and seven alleles appeared with frequencies of > 5% in the Baggarar, Kenana and Butana breeds, respectively. These common alleles accounted for a low proportion of the cumulative gene frequencies (12.83, 44.23 and 50.83% in the Baggara, Kenana and Butana breeds, respectively); four of which (BoLA-DRB3*003:02:01, *021:01 *022:01 and *024:01) were common in at least two out of the three Sudanese breeds (Table 1).

### Nucleotide and amino acid diversity in the *BoLA-DRB3* alleles found in native Sudanese cattle breeds

Genetic diversity at the DNA and amino acid levels was evaluated using four methods that compare the average amino acid and nucleotide substitutions for every pair of alleles within the breeds. The nucleotide diversity (π) exceeded 0.074 and the mean number of pairwise differences values exceeded 17.99 within Sudanese native breeds (Table 3). Comparison with results previously reported for other cattle breeds showed that these nucleotide diversity values all fall within the range previously reported (π_range_ = 0.068–0.090; NPD_range_ = 16.31–20.96) when using PCR-SBT genotyping methods^18,19,21,29,30^. Regarding amino acid diversity, the average d_N_ and d_S_ substitutions in Sudanese cattle breeds were calculated across the entire *BoLA-DRB3* exon 2 and ABS. As expected, the d_N_/d_S_ ratio was higher when only the ABS was analyzed (Table 3). These values obtained in Sudanese cattle were similar to those estimated for other cattle breeds (d_N_/d_S total_ = 3.50–3.85; d_N_/d_S ABS_ = 4.80 – 4.93).

**Table 3:** Nucleotide diversity (π), mean number of pairwise differences (NPD) and mean number of non-synonymous (d_n_) and synonymous (d_s_) nucleotide substitutions per site.

Breed	π	NPD	Total			ABS		
			ds	dn	dn / ds	ds	dn	dn / ds
Baggara	0.080	19.36	0.030	0.105	3.50	0.090	0.435	4.83
Butana	0.074	17.99	0.028	0.105	3.75	0.093	0.447	4.80
Kenana	0.075	18.28	0.027	0.104	3.85	0.090	0.444	4.93

### Gene diversity, Hardy–Weinberg Equilibrium (HWE), and neutrality testing of *BoLA-DRB3* variants found in Sudanese cattle breeds

**Figure 4:**
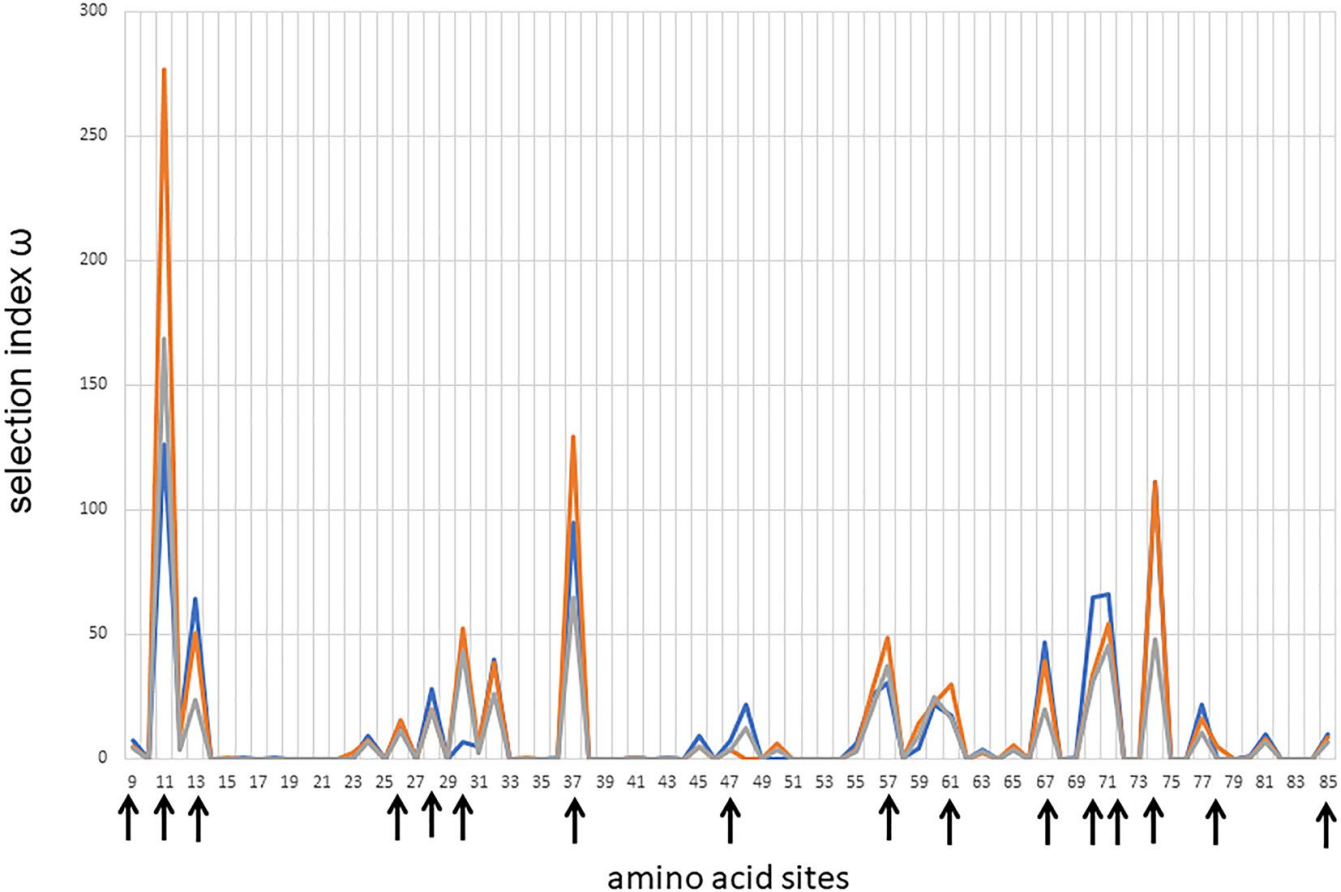
Estimated values of the selection index ω in each amino acid site along *BoLA-DRB3* exon 2 in Baggara (grey), Kenana (blue) and Butana (brown) Sudan cattle breeds. Arrows indicate the antigen-binding site (ABS).

Genetic diversity within the three Sudanese breeds was estimated using the n_a_ and gene diversity (h_o_ and h_e_). We also performed HWE and Slatkin´s neutrality tests on *BoLA-DRB3* to evaluate the possible effect of selection, inbreeding, and population structure on allelic diversity at this locus. The high n_a_ values and even gene frequencies observed in the Butana, Kenana and Baggara breeds resulted in h_e_ and h_o_ values higher than 0.93 (Table 2). As expected, these indices highlighted extremely high diversity values for Sudanese cattle populations, which is similar to the results reported for other bovine breeds which have been evaluated by PCR-SBT, and characteristic of *MHC class II DR* genes^18,19,21,27,29,35^. Regarding the HWE test, the three Sudanese native populations were in equilibrium (Table 2), similar to observations in half of the bovine breeds studied so far. It is widely accepted that the genetic diversity of *MHC class II* genes can be maintained by balancing selection. Thus, we performed a Slatkin’s exact neutrality test (Table 2) to evaluate this phenomenon in the Sudanese cattle populations. The *BoLA-DRB3* gene frequency profile in Baggara cattle showed an even distribution (*p* = 0.016), consistent with the theoretical proportion expected under balancing selection pressures. A similarly even *BoLA-DRB3* gene frequency was observed in other cattle breeds, including Japanese Black, Yacumeño Creole, Bolivian Gir, Pyer Sein and Shwe Ni. Conversely, we did not detect balancing selection in the Butana and Kenana cattle (*p* = 0.225 and *p* = 0.138) despite these breeds having a large number of alleles with similar frequency. Comparable results were obtained for the majority of the cattle breeds analyzed to date (Table 2). In addition, we estimated the selection index (ω) in each amino acid site to evaluate the presence of diversifying selection (ω > 1) along *BoLA-DRB3* exon 2. These analyses showed high ω values in more than 30 sites in each breed, mainly located in the ABS (Fig. 4).

### BoLA-DRB3 genetic structure and levels of population differentiation in Sudanese cattle

**Figure 5:**
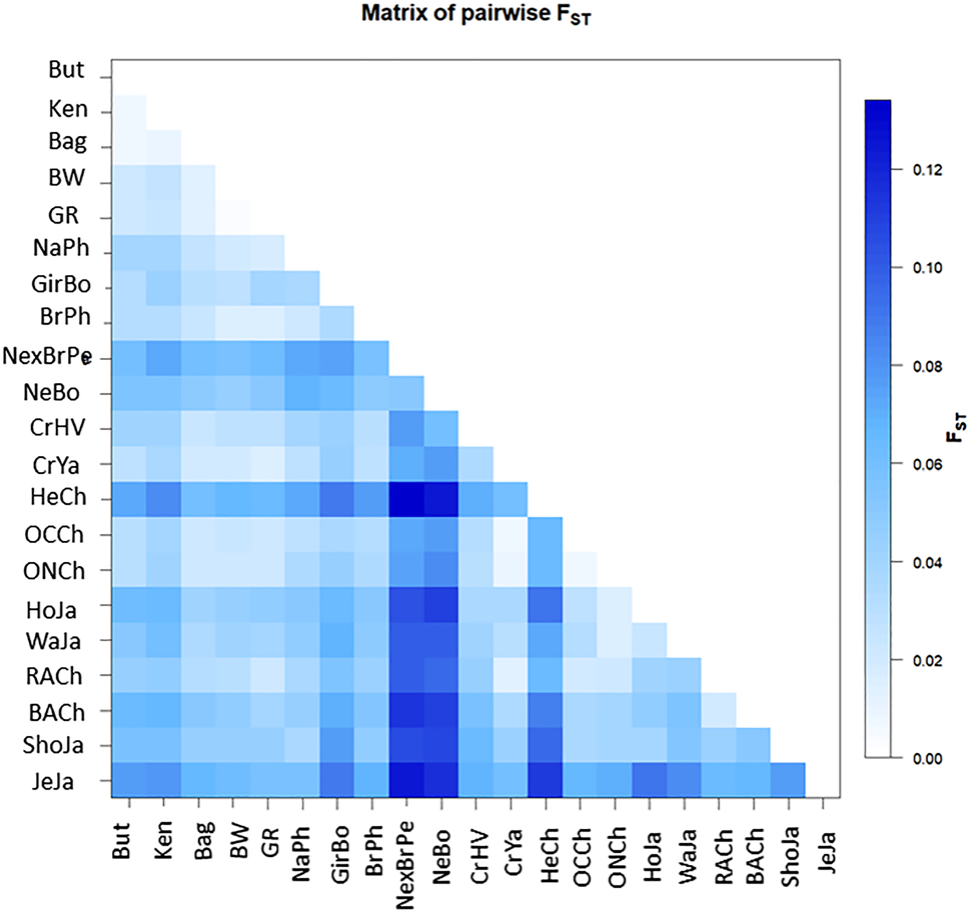
Graphic representation of calculated F_ST_ between population pairs using an R package pairFstMatrix.r. But = Butana, Ken = Kename, Bag = Baggara, BW = Pyer Sein. GR = Shwe Ni, NaPh = Philippine native, GirBo = Bolivian Gir, BrPh = Philippine Brahman, BrxNePe = Peruvian Brahman × Nellore crossbreed, NeBo = Bolivian Nellore, CrHV = Creole Hatón del Valle, CrYa = Creole Yacumeño, HeCh = Chilean Hereford, OCCh = Chilean Overo Colorado, ONCh = Chilean Overo Negro, HoJa = Japanese Holstein, WaJa = Japanese Black, BACh = Chilean Black Angus, RACh = Chilean Red Angus, ShJa = Japanese Shorthorn and JeJa = Japanese Jersey.

The level of genetic differentiation among the three Sudanese breeds was studied through the *F*_ST_ index. The average *F*_*ST*_ was statistically significant although this value accounts for less than one percent of the total genetic variance (*F*_*ST*_ = 0.0076 (ranging between 0.007 and 0.009); *p* < 0.001) (Table S2). This low but significant value can be explained by high within-population diversity and differences in rare alleles profiles among them^36^. The average *F*_*ST*_ value observed in Sudanese cattle is higher than those estimated in Myanmar native breeds (*F*_*ST*_ = 0.003), and slightly lower than those reported for Holstein populations from different countries (*F*_*ST*_ = 0.009)^18,37^ (Fig. 5 and Table S2). When breeds were grouped in terms of the breed’s geographical origin, as was done in the Venn diagram, the genetic variance among breed groups and among populations within groups accounted for 1.18% and 3.71% of the total genetic variance. Table S2 summarizes the genetic distance, measured by *F*_ST_**,** between native Sudanese breeds and other taurine and zebu breeds for *BoLA-DRB3*, showing that native Sudanese cattle diverge from other breeds with *F*_*ST*_ values between 0.014 and 0.082.

When the five sampling sites of native Sudanese breeds were compared (two sampling locations of Kenana cattle were very close and assumed as one), the average *F*_ST_ value was 0.0074 (*p* = 0.164), while the pairwise *F*_ST_ ranged from 0.0002 (*p* = 0.450) between both Baggara populations and 0.0118 (*p* < 0.0001) between Baggara Daiwani and Butana Qadarif. Significant differences were observed in nine out of the ten native population comparisons (*p* < 0.05; Table S3). Similar genetic distance values were observed among Holstein populations from different countries and between native breeds of Myanmar^18,37^.

### Genetic differentiation of *BoLA-DRB3* alleles in native Sudanese cattle breeds: comparison with Zebu and Taurine breeds

**Figure 6:**
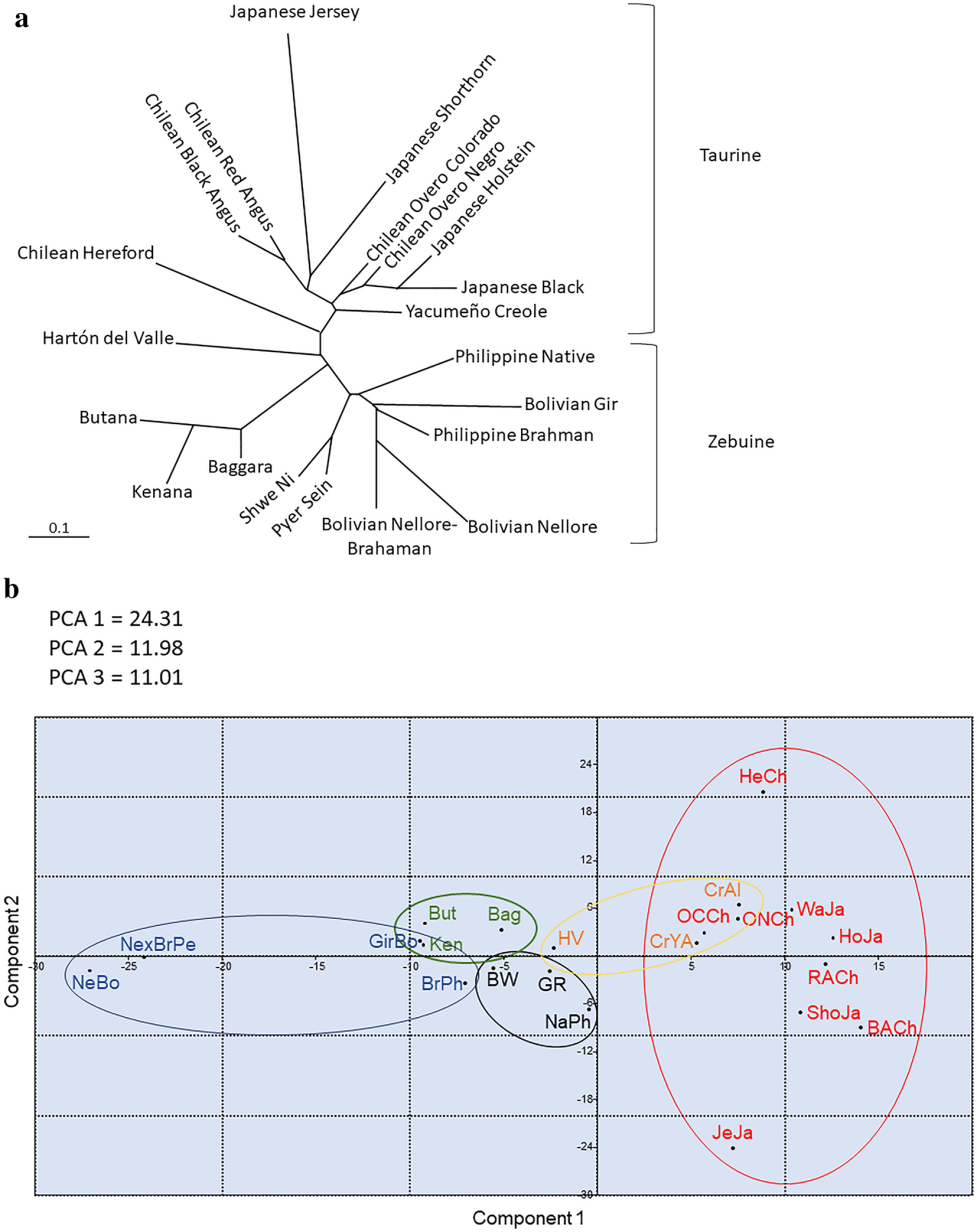
(**a**) Neighbor-joining dendrogram constructed from a matrix of D_A_ genetic distances. (**b**) Principal Component Analysis of allele frequencies from the BoLA-DRB3 gene in 22 breeds. But = Butana, Ken = Kename, Bag = Baggara, BW = Pyer Sein. GR = Shwe Ni, NaPh = Philippine native, GirBo = Bolivian Gir, BrPh = Philippine Brahman, BrxNePe = Peruvian Brahman × Nellore crossbreed, NeBo = Bolivian Nellore, CrHV = Creole Hatón del Valle, CrYa = Creole Yacumeño, Creole Highland, HeCh = Chilean Hereford, OCCh = Chilean Overo Colorado, ONCh = Chilean Overo Negro, HoJa = Japanese Holstein, WaJa = Japanese Black, BACh = Chilean Black Angus, RACh = Chilean Red Angus, ShJa = Japanese Shorthorn and JeJa = Japanese Jersey.

First, *BoLA-DRB3* allele frequencies from Sudanese cattle populations and for each breed included in the dataset were used to generate Nei’s D_A_ and D_S_ genetic distance matrices. Then, dendrograms were constructed from these distance matrices using NJ algorithm. All trees revealed congruent topologies, which were consistent with the historical and geographical origin of the breeds analyzed. As expected, these trees revealed two main clusters, which included the Taurine and Zebuine breeds (Fig. 6a). It is noteworthy that Sudanese breeds were located in a sub-cluster within the indicine cluster, with the two dairy breeds located in the east of the country, Butana and Kenana being more related to each other than the Baggara breed in the west. These results reveal that Sudanese cattle breeds have a particular diversity in the *BoLA-DRB3* gene, as a consequence of its gene frequency profile and the presence of a high number of private alleles.

The results of the PCA showed that the first three components accounted for 47.30% of the data variability. The first principal component (PC) accounted for 24.31% of the total variance and, as shown in a previous study^64^, clearly exhibited a differentiation pattern between the Zebu (negative values) and Taurine (positive values) breeds, while native breeds from Southeast Asia and Sudan were located in an intermediate position near the axis origin of the plot (Fig. 6b). This PC was primarily determined by differences in the frequency of the same alleles, such as BoLA-DRB3*022:01, *028:01, *036:01, *031:01, *030:01, and *057:02 with the higher negative axis 1 values, whereas the alleles BoLA-DRB3*001:01, *002:01, *007:01, *008:01, *010:01, *011:01, *012:01, *015:01 *016:01, *018:01 had the higher positive values for this axis. The second PC explained 11.98% of the total variation and showed a gradient among Taurine breeds, with Chilean Hereford (positive values) and Japanese Jersey (negative values) located at opposite ends. Furthermore, this component discriminated between native Sudanese and native Southeast Asian cattle breeds. Finally, the third PC accounted for 11.01% of the variance and allowed for the differentiation of Chilean Hereford, and Japanese Jersey and Japanese Holstein cattle from other Taurine breeds. In summary, the native Sudanese cattle breeds were located within a narrow cloud in an intermediate position between the Zebu and Taurine breeds and close to other Southeast Asian breeds, in agreement with the composite origin of these native breeds. This is also supported by the presence of African and Zebu unique *BoLA-DRB3* alleles within these populations. These PCA results agree with the overall clustering observed after NJ tree construction.

The BoLA class II molecule binds peptides derived from antigens via five antigen binding pockets named pocket 1, pocket 4, pocket 6, pocket 7 and pocket 9^24^. To assess whether observed differences in allelic frequency are reflected within amino acid motifs in each pocket, we analyzed frequency of the protein pockets implicated in the antigen-binding function of the MHC complex by PCA. As shown in Fig. S3a-e, the three native breeds of Sudan are located in a closed cloud in the five PCAs made based on the frequency of the pockets, although varying their relative position with other breeds and breed groups, and in some cases the spatial distribution did not exhibit a clear relationship with the geographical or historical origin of the breeds. However, pockets 4 and 9 are the ones that best differentiate these native breeds from the rest. Regarding pocket 4, Baggara and Kenana breeds of Sudan are located in a narrow cloud located at the end of axis 2, and their position is mainly explained by the GFDEREY, RFDERFV and GLDRKEV motifs. The position of the Butana and Kenana Sudanese breeds in pocket 9 was the result of positive PC1 and PC2 values for the presence of amino acid motifs EYD and EFA.

**Figure 7:**
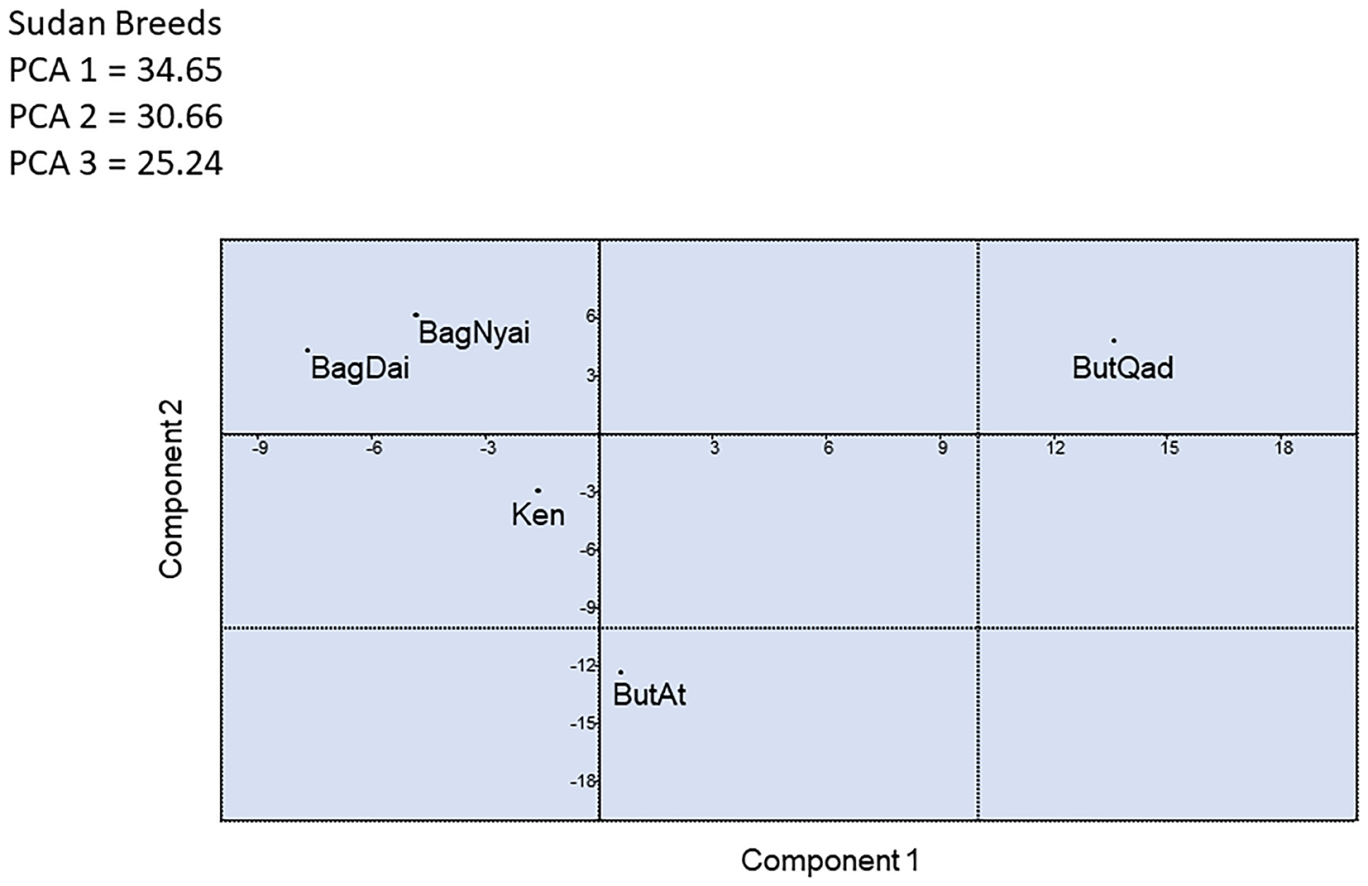
Principal components analysis of allele frequencies from the BoLA-DRB3 gene in five Sudan native samples sites (BagDai = Baggara Daiwani, BagNyai = Baggara Nyakawi, Ken = Kenana, ButAt = Butana Bu Atbara, and ButQad = Butana Bu Qadarif).

Finally, PCA was performed at the Sudanese population level to evaluate the degree of genetic structure among the sampling sites (Baggara Daiwani, Baggara Nyakawi, Kenana, Butana Bu Atbara and Butana Bu Qadarif). This analysis showed that the first three components accounted for 90.95% of the data variability. The first PC accounted for 30.65% of the total variance and clearly exhibited a differentiation pattern between the Baggara population (negative values) and the Butana Bu Qadarif (positive values) population, while Kenana, Butana Bu Atbara were located in intermediate positions (Fig. 7). These results agree with the geographical distribution of the studied population. The second and third PCs explained 30.66% and 25.24% of the total variation and allowed for the differentiation of the Butana Qadarif and Kenana populations, respectively.

## Discussion

Since the first pioneering studies based on serotype analysis, a number of striking differences between the BoLA profiles of African and European cattle have been reported due to difference in the antigen’s frequency of occurrence and the presence of unique antigens in African cattle^38^. Over the next decades, several private alleles were identified in taurine, zebu and taurindicus native African breeds, like N´Dama, Boran, and Sanga^32,34^; https://www.ebi.ac.uk/ipd/mhc/group/BoLA/). However, in the present study, we carried out the first genetic characterization of the *BoLA-DRB3* gene at population level in native Sudanese breeds using PCR-SBT. This analysis allowed us to detect 53 alleles, including seven new variants. The high number of private alleles agrees with data obtained by^16^, who analyzed the BoLA region in depth using a genome-wide sequencing approach, identifying six major African BoLA haplotype blocks.

Wild cattle or ‘aurochs’ (*Bos primigenius*), the ancestor of domestic cattle, inhabited a large geographical area throughout Eurasia and North Africa. According to the trans-species theory of MHC alleles^39^, it is expected that the extremely high genetic variability present in the *BoLA-DRB3* gene (365 alleles have been reported in the IPD-MHC (https://www.ebi.ac.uk/ipd/mhc/group/BoLA;^33^ database, access date 16/04/21) was present in the wide geographical distribution of the aurochs. On the basis of archeological and genetic studies, it has been proposed that modern bovines were domesticated in two geographical sites, one located in the West Asia (Near east), and the other in Indian subcontinent (India and Pakistan)^40–46^. Each of these domestication centers would have retained only a fraction of the total diversity as a result of bottleneck and genetic drift effects^47^. This is clearly seen in the distribution of mitochondrial haplogroups among cattle breeds^5,40–44^. In Africa, taurine cattle originated from the Near east domestication center, and introgressed through the North part of the continent and from there they would have dispersed east, west and south. Then, indicine cattle were introduced to Africa and *Bos indicus* genes were introgressed into native populations through absorbent crosses^48^. Currently, an east–west gradient of Zebu influence in African native genes is observed.

Subsequent dispersal and crossbreed processes described above (founder group, migration and gene introgression) and natural and artificial selection would have shaped the *BoLA-DRB3* diversity in the current bovine populations. Accordingly, the *BoLA-DRB3* alleles detected in the Sudanese cattle were interspersed distributions along the allele NJ tree instead of grouped in specific clusters of the dendrogram, which is consistent with the ancient origin of the *BoLA-DRB3* alleles. Similar results have been reported in other native cattle breeds from different geographical regions^21,22^.

Our Venn diagram illustrates the distribution of allelic diversity among different bovine groups, demonstrating that 14 *BoLA-DRB3* alleles were only detected in the Sudanese cattle breeds. Seven of these alleles corresponded to new variants described in this study (Table 1). Furthermore, a review of the IPD–MHC database showed that this group of Sudanese private alleles included seven other variants previously detected only in African breeds (Table S4).

Two *BoLA-DRB3* alleles, that were only previously reported in Creole cattle breeds^21,37^, were identified in native Sudanese breeds. Studies based on mitochondrial DNA and Y chromosome haplotypes have revealed an African component in the germplasm of the American creole bovine breeds. Two origins have been proposed for this African component: through the native Iberian cattle that are the ancestors of Creole cattle and/or a direct introgression from mainland Africa following the slave trade routes^49^. The Iberian theory is unlikely as the BoLA-DRB3*011:02 and BoLA-DRB3*029:02 alleles have not been detected in the Spanish Morucha breed, which were only autochthonous Iberian breed in which the genetic diversity of the *BoLA-DRB3* gene has been studied so far^20^. In summary, 16 possible African putative alleles were identified in the native bovine populations of Sudan, totaling 20.22% of the gene frequency. The presence of private *BoLA-DRB3* alleles (not detected in zebu breeds so far) in native African breeds with humped phenotype suggest that current global diversity of this gene could have been retained in the founder group that originate African taurine native breeds^45^.

On the other hand, a group of alleles is shared between the Sudanese breeds and the Zebu, Southeast Asian and/or Creole American breed groups (Table S5), but is absent in the European breeds. It is worth noting that these alleles were first identified in cattle breeds such as Boran, Ethiopian Arsi, N´Dama and Brahman (^32,34,50^; https://www.ebi.ac.uk/ipd/mhc/group/BoLA/) (Table S5). The introgression of these variants could have been a consequence of the successive waves of introduction of Zebu cattle into the African continent^48^. These alleles account for an additional 15.33% of the gene frequencies. The remaining alleles have a worldwide geographical distribution; thus, 20 variants have been detected in all the breed groups included in the Venn diagram. Further studies on the genetic diversity of the *BoLA-DRB3* gene in other African bovine populations will surely reveal a greater allelic repertoire.

The current repertoire of alleles of the *BoLA-DRB3* gene in the native cattle of Sudan would not only have been molded by stochastic forces, such as the formation of the founder group, gene drift and recent or historical gene introgression as described above, but also by processes of natural and artificial selection. In Sudan, as in other African regions, cattle are subjected to strong environmental pressures, such as tropical diseases, heat stress, drought and poor nutritional and forage deficits. Furthermore, animals are affected by diverse infectious diseases, including parasites (e.g., ticks, theileriosis, babesiosis, anaplasmosis, trypanosomosis;^51–57^, bacteria (e.g., Hemorrhagic septicemia, Anthrax, tuberculosis, brucellosis, Thrombotic meningoencephalitis;^58–62^) and viruses (e.g., foot and mouth disease, lumpy skin disease, Pox virus, bovine viral diarrheal diseases complex;^53,63,65^). For this reason, it is to be expected that native Sudanese cattle will be under strong selection pressure, which would contribute to maintaining and shaping the genetic diversity of the *BoLA-DRB3* gene. In this sense, a wide repertoire of alleles allows the population to identify and respond to a greater range of antigens. Furthermore, heterozygous animals trigger an immune response to a greater variety of antigens. For these reasons, it has been proposed that this allelic diversity is maintained by balancing or over-dominant selection^30,65,66^. Different indices at the population, nucleotide and amino acid levels showed high levels of genetic diversity in the bovine breeds of Sudan for the *BoLA-DRB3* gene. This is clearly reflected in the presence of a homogeneous distribution of gene frequencies (a high number of alleles with low frequencies). This is particularly extreme in the Baggara breed in which Slatkin’s neutrality test showed evidence that the *BoLA-DRB3* gene frequency profile showed an even distribution consistent with the theoretical proportion expected under balancing selection pressures. Similar results have been reported for other cattle breeds, including Japanese Black, Yacumeño Creole, Bolivian Gir, Pyer Sein and Shwe Ni^21,22,30^. Furthermore, the selection index (ω) revealed the presence of diversifying selection in several amino acid sites (mainly in the ABS) in *BoLA-DRB3* exon 2 of the Sudan native breeds. In contrast, the HWE test did not detect the effect of over-dominant selection^67^. As discussed previously^21^, this effect has been observed only in some of the breeds studied so far and the most common explanation for the absence of heterozygote excess in the studied bovine breeds is the magnitude of the overdominance selection coefficient at MHC loci (probably lower than 0.02;^68^). Such selection would only be enough to increase the number of heterozygotes in large populations and in the absence of high rates of stochastic forces (population bottlenecks, genetic drift, and inbreeding). For this reason, and because the HWE method may suffer from low resolving power, such effects were not observed.

The repertoire of alleles of the *BoLA-DRB3* gene present in the native cattle of Sudan allows these breeds to be clearly differentiated from the rest, forming a cluster in the NJ trees and a narrow cloud in the PCA. This pattern is confirmed when PCAs are performed based on the pocket 4 and pocket 9 gene frequencies. It has previously been proposed that pocket 4 plays an important role in the binding of peptides due to this pocket being located in the center of the PBC 64^69,70^. In addition, it has been reported in cattle that immune responses against vaccine and disease resistance is significantly related to differences in the pocket 4 motif^49,50^. A particular amino acid (e.g., amino acid R in position 70) or amino acid motifs (e.g., ER at 70 and 71 sites; EIAY motif at positions 66–67–74–78, and the deletion of the amino acid 65), in sites that affect the conformation of pocket 4, have been associated with immune response or resistance to infectious diseases, such as mastitis, persistent lymphocytosis, dermatophilosis, and tick-borne diseases^25,50,69,71–73^. Many of these diseases, as well as others mentioned above, are present in Sudan and could have contributed to shaping the current repertoire of *BoLA-DRB3* alleles present in native Sudanese cattle. However, these results were obtained in breeds that have different genetic backgrounds and that are raised in different environments and production systems, so further association studies are necessary to determine the effect (resistance or susceptibility) of the alleles present in the native cattle breeds of Sudan against different infectious diseases.

## Conclusions and future prospects

To the best of our knowledge, this is the first study to document in detail the genetic diversity (taurine vs indicine) of *BoLA-DRB3* alleles in cattle not only in Sudan but in the entire African continent. In addition to the clear genetic clustering of cattle based on ancestral origin and phylogeography, we identify seven novel alleles in the three native Sudanese cattle breeds. Two evolutionary forces appear to contribute to the preservation and shaping of the genetic diversity of the *BoLA-DRB3* gene in native Sudanese cattle; diversifying selection mainly affects the ABS of the native breeds and balancing selection. The results demonstrate that the background variation between two cattle groups, taurine and indicine, is primarily due to events of origin, selection, and adaptation, which explains the variations found in the diversity of the *BoLA-DRB3* genes, not only between the two major groups but also with the indicine cattle group. This variation may explain how cattle from Sudan are resistant to various diseases. We presume that this genetic information provides a basis for better design of suitable breeding schemes. This variation may contribute to resistance in Sudanese cattle to various diseases.

## Materials and methods

### Sampled populations and genomic DNA extraction

The ODK (Open Data Kit) system was used to record the sampling information: breed name, sex, estimated age, sampling location GPS coordinates, photo of the animal and owner’s information. All methods were carried out in accordance with relevant guidelines and regulations of the Faculty of Veterinary Medicine, University of Khartoum (Vet. Med. U of K), and all experimental protocols were approved by the Vet. Med. U of K research board committee. Before animals were sampled, written informed consents were obtained from all animal owners. Three cattle breeds were examined: (1) Butana breed: collected from the Atbara Butana Station and surrounding villages and from El-Gadarif city and Butana plain; (2) Kenana breed: samples were collected from Rabak city and surrounding villages and from UmBanein Kenana Station; (3) Baggara breed populations (i) Nyalawi population, which is a western Baggara breed sampled from calves from Nyala city, South Darfur; (ii) Daeinawi population, from Ed daein city. Whereas Nyalawi are large white cattle, some with black splashes, the Daeinawi are smaller and red with black along the neck and lateral sides of the head, hind quarters and shoulder sides (Fig. S4).

A total of 225 native breed cattle were sampled: Baggara N = 113, Butana N = 60 and Kenana N = 52 (Table S1 and Fig. S4). Seven milliliters of venous blood were collected in EDTA-containing vacutainer tubes. Genomic DNA was extracted using DNeasy® Blood and Tissue Kit, (Qiagen, Germany), following the manufacturer’s instructions.

### PCR amplification and sequencing

Exon 2 of the *BoLA-DRB3* was amplified by PCR as described by^26^. Using DRB3FRW 5-CGCTCCTGTGA(C/T)CAGATCTATCC-3 and DRB3REV 5-CACCCCCGCGCTCACC-3, PCR reactions were performed in a 25 μl-reaction mixture containing 12.5 μl of 2× Gflex PCR Buffer (Mg^+2^, dNTP plus) (TaKaRa Bio Inc., Shiga, Japan), and 0.5 μl of Tks Gflex DNA polymerase (1.25 units/μl) (TaKaRa Bio Inc.), 200 nM of each primer, and 1.0 μl of template. The reaction conditions consisted of an initial denaturation step at 95 °C for 3 min, followed by 35 cycles of 95 °C for 1 min, 58 °C for 30 s and 68 °C for 90 s and a final extension step at 68 °C for 5 min. PCR products were purified using a NucleoSpin Gel and PCR Clean Up Kit (Takara Bio Inc.). Cycle sequencing reactions were performed directly using the two PCR primers using the BigDye Terminator version 3.1 Cycle Sequencing Kit (Applied Biosystems, Foster City, CA, USA) and analyzed on an ABI Prism 3130 × genetic analyzer (Applied Biosystems) according to the manufacturer’s instructions.

### Sequence data analysis

Prior to analysis, all the chromatograms were visualized and sequence fragments were edited manually using ATGC software version 9.1 (GENETYX Corporation, Tokyo, Japan) correcting base calling errors. Multiple sequence alignments were performed using the MUSCLE algorithm implemented in MEGA X^74^, and were subsequently joined to reconstruct a fragment of 280 bp spanning the entire exon 2.

### BoLA-DRB3 allele genotyping

For typing *BoLA-DRB3* genotypes, we used the method implemented by^26^: First, we downloaded a MHC_nuc.txt file from the IPD-MHC in order to update the allele database. This file contains all reported BoLA-DRB3 alleles. Then DNA sequences from the cattle for both strands (forward and reverse ab1 files) were imported together into the Assign 400ATF ver. 1.0.2.45 software (Conexio Genomics, Fremantle, Australia), which automatically aligned the sampled cattle sequences with those of previously reported *BoLA-DRB3* sequences, building a consensus. The most likely genotype is shown in the same window as the chromatograms so that they can be crosschecked. When we found a clear mismatch from several samples, we assigned these samples containing new alleles and revised the *BoLA-DRB3* database containing new allele sequences. The accuracy of the in silico genotyping method was demonstrated in Takeshima et al. (2001, 2011) where the new detected alleles were confirmed by cloning and sequencing, and the used method was developed and validated for only the *BoLA-DRB3* gene. If the sample could not genotype using these criteria, we discarded the sample result from this analysis.

### Statistical analyses

#### Genetic diversity at allele level

Allele frequencies and the number of alleles (n_a_) were obtained by direct counting. The distribution of alleles across breeds was analyzed by a Venn plot created using the R package ‘VennDiagram’ (http://cran.r-project.org/). The observed (h_o_) and unbiased expected (h_e_) heterozygosity of the *BoLA-DRB3* locus were estimated according to^73^ using the Arlequin 3.5 software for population genetic analyses^76^ (Schneider, 2000). *F*_IS_ statistics^77^ for each breed were calculated using the Exact Test included in Genepop 4.7 software^78^ to evaluate deviation from Hardy–Weinberg equilibrium (HWE). The Ewens–Watterson–Slatkin Exact Test of neutrality was carried out using the method described by^79^ and implemented in the Arlequin 3.5 program.

#### Breed genetic structure

Genetic structure and genetic differentiation within Sudanese cattle breeds and among bovine breeds were assessed using Wright's *F*_ST_ statistics^77^. This parameter was estimated using Arlequin 3.5 and Genepop 4.7 software. The *F*_ST_ values were represented graphically using the pairFstMatrix.r function implemented in the R statistical environment.

#### Genetic relationship between breeds

To condense the genetic variation at the *BoLA-DRB3* locus, allele frequencies were used to perform a PCA according to the^80^ method, implemented in Past software^81^. Nei's standard genetic distances Ds^82,83^ were calculated from allele frequencies and were used to perform cluster analysis using the Neighbor-Joining (NJ) algorithm^84^. Confidence intervals for the groupings were estimated by bootstrap resampling of the data using 1000 replicates. Genetic distances and trees were computed using the Populations 1.2.28 software ^84^. The trees were then visualized using TreeView^85^.

#### Genetic diversity at sequence level

Nucleotide diversity (π) and pairwise differences in nucleotide substitutions between alleles within each breed were calculated using Arlequin 3.5. The mean number of nonsynonymous (d_N_), and synonymous (d_S_) nucleotide substitutions per site from averaging over all sequence pairs were estimated within each group using the modified Nei-Gojobori model^83^ and Jukes–Cantor’s formula implemented in the software MEGA X^72^. The possibility that certain codon sites are under diversifying selection within each native Sudan breed was investigated using the Bayesian method implemented using OmegaMap^86^. This method incorporates intragenic recombination and does not assume a known fixed genealogy, so that recombination does not inflate the false detection rate of positive sites^87^. The *BoLA-DRB3* allele tree was constructed from a distance matrix that was based on the NJ method using the MEGA X software. Furthermore, a tree based only on ABS amino acid motifs was inferred using Maximum Parsimony method implemented in MEGA X. To test the significance of the branches of both trees, 1000 bootstrap replicate calculations were performed.

## Supplementary Information

Supplementary Information.

## Data Availability

Supplementary Material contains Table S1-S5 and Figures S1-S3 including detailed descriptions of all supplemental files.
