# Supplementary material for: BoLA-DRB3 gene haplotypes show divergence in native Sudanese cattle from taurine and indicine breeds
Source: Sci Rep. 2021 Aug 25;11:17202. doi: 10.1038/s41598-021-96330-7 (PMC8387388; doi:10.1038/s41598-021-96330-7)

**Supporting information**

**BoLA-DRB3 gene haplotypes show divergence in native Sudanese cattle from Taurine and Zebu breeds**

Bashir Salim*^1^, Shin-nosuke Takeshima^2^, Ryo Nakao^3^, Mohamed AM Moustafa^3^, Mohamed-Khair A. Ahmed^4^, Sumaya Kambal^5^, Joram M. Mwacharo6, Guillermo Giovambattista^7^

**Table S1** Detailed information about the populations analyzed of indigenous Sudan cattle breeds

| **Acronym** | **Sample size** | **Breed** | **Type** | **Strain** | **Production purpose** | **Origin (country)** | **Sampling country** | **Geographical position** |
| --- | --- | --- | --- | --- | --- | --- | --- | --- |
| Bag | 113 | Baggara | Zebu | Daiwani (N = 56)  Nyalawi (N = 57) | beef | Sudan, Niger, Chad, Cameroon, Nigeria, Central African Republic. | Sudan | 11.4610454,26.0983731  12.001278,24.8629579 |
|  |  |  |  |  |  |  |  |  |
| But | 60 | Butana | Zebu | Atbara (N = 32)  Qadarif (N = 35) | dairy | Sudan | Sudan | 17.6923089,33.976163  14.0318311,35.3065785 |
|  |  |  |  |  |  |  |  |  |
| Ken | 52 | Kenana | Zebu | Rabak (N= 29)  UmBanein (N= 23) | dairy | Sudan | Sudan | 13.1811485,32.7073246  13.1448529,33.9091384 |

**Supporting information**

**Table S2.** Genetic distance between pair of breeds/populations estimated through Nei DA distance (above) and F_ST_ (below). But = Butana, Ken = Kename, Bag = Baggara, BW = Pyer Sein. GR = Shwe Ni, NaPh = Philippine native, GirBo = Bolivian Gir, BrPh = Philippine Brahman, BrxNePe = Peruvian Brahman × Nellore crossbreed, NeBo = Bolivian Nellore, CrHV = Creole Hatón del Valle, CrYa = Creole Yacumeño, HeCh = Chilean Hereford, OCCh = Chilean Overo Colorado, ONCh = Chilean Overo Negro, HoJa = Japanese Holstein, WaJa = Japanese Black, BACh = Chilean Black Angus, RACh = Chilean Red Angus, ShJa =Japanese Shorthorn and JeJa = Japanese Jersey.

|  | But | Ken | Bag | BW | GR | NaPh | GirBo | BrPh | NexBrPe | NeBo | CrHV | CrYa | HeCh | OCCh | ONCh | HoJa | WaJa | RACh | BACh | ShoJa | JeJa |
| --- | --- | --- | --- | --- | --- | --- | --- | --- | --- | --- | --- | --- | --- | --- | --- | --- | --- | --- | --- | --- | --- |
| But | 0.000 | 0.213 | 0.225 | 0.560 | 0.599 | 0.727 | 0.533 | 0.545 | 0.596 | 0.549 | 0.641 | 0.588 | 0.685 | 0.622 | 0.558 | 0.761 | 0.708 | 0.680 | 0.728 | 0.793 | 0.907 |
| Ken | 0.007 | 0.000 | 0.239 | 0.580 | 0.609 | 0.670 | 0.606 | 0.531 | 0.607 | 0.584 | 0.635 | 0.608 | 0.784 | 0.617 | 0.639 | 0.727 | 0.750 | 0.699 | 0.729 | 0.761 | 0.902 |
| Bag | 0.007 | 0.009 | 0.000 | 0.468 | 0.517 | 0.584 | 0.550 | 0.451 | 0.535 | 0.508 | 0.454 | 0.433 | 0.632 | 0.448 | 0.438 | 0.563 | 0.516 | 0.525 | 0.623 | 0.650 | 0.781 |
| BW | 0.021 | 0.026 | 0.014 | 0.000 | 0.164 | 0.313 | 0.443 | 0.241 | 0.383 | 0.384 | 0.535 | 0.397 | 0.673 | 0.453 | 0.426 | 0.559 | 0.540 | 0.475 | 0.581 | 0.618 | 0.779 |
| GR | 0.021 | 0.025 | 0.014 | 0.003 | 0.000 | 0.356 | 0.569 | 0.291 | 0.430 | 0.434 | 0.540 | 0.402 | 0.631 | 0.475 | 0.411 | 0.570 | 0.553 | 0.467 | 0.549 | 0.645 | 0.788 |
| NaPh | 0.038 | 0.038 | 0.027 | 0.019 | 0.018 | 0.000 | 0.481 | 0.248 | 0.453 | 0.524 | 0.576 | 0.506 | 0.685 | 0.479 | 0.534 | 0.593 | 0.558 | 0.547 | 0.556 | 0.562 | 0.681 |
| GirBo | 0.033 | 0.043 | 0.031 | 0.028 | 0.039 | 0.037 | 0.000 | 0.390 | 0.466 | 0.481 | 0.591 | 0.577 | 0.706 | 0.561 | 0.627 | 0.695 | 0.780 | 0.719 | 0.682 | 0.852 | 0.890 |
| BrPh | 0.032 | 0.033 | 0.024 | 0.015 | 0.015 | 0.022 | 0.035 | 0.000 | 0.306 | 0.323 | 0.475 | 0.398 | 0.650 | 0.462 | 0.492 | 0.591 | 0.583 | 0.559 | 0.581 | 0.605 | 0.755 |
| NexBrPe | 0.060 | 0.073 | 0.059 | 0.057 | 0.062 | 0.073 | 0.074 | 0.058 | 0.000 | 0.336 | 0.519 | 0.431 | 0.753 | 0.446 | 0.470 | 0.570 | 0.668 | 0.662 | 0.730 | 0.715 | 0.819 |
| NeBo | 0.056 | 0.055 | 0.050 | 0.045 | 0.052 | 0.068 | 0.063 | 0.049 | 0.051 | 0.000 | 0.486 | 0.608 | 0.781 | 0.673 | 0.665 | 0.823 | 0.759 | 0.825 | 0.791 | 0.898 | 0.878 |
| CrHV | 0.041 | 0.041 | 0.024 | 0.029 | 0.029 | 0.038 | 0.042 | 0.030 | 0.076 | 0.060 | 0.000 | 0.451 | 0.540 | 0.462 | 0.464 | 0.492 | 0.538 | 0.578 | 0.565 | 0.745 | 0.694 |
| CrYa | 0.028 | 0.036 | 0.019 | 0.019 | 0.015 | 0.029 | 0.044 | 0.028 | 0.071 | 0.077 | 0.035 | 0.000 | 0.469 | 0.202 | 0.208 | 0.309 | 0.355 | 0.236 | 0.356 | 0.465 | 0.648 |
| HeCh | 0.072 | 0.082 | 0.059 | 0.066 | 0.064 | 0.072 | 0.088 | 0.077 | 0.134 | 0.124 | 0.071 | 0.059 | 0.000 | 0.470 | 0.476 | 0.548 | 0.539 | 0.516 | 0.535 | 0.702 | 0.801 |
| OCCh | 0.031 | 0.038 | 0.021 | 0.024 | 0.022 | 0.029 | 0.037 | 0.033 | 0.072 | 0.077 | 0.033 | 0.008 | 0.063 | 0.000 | 0.134 | 0.243 | 0.324 | 0.215 | 0.307 | 0.325 | 0.679 |
| ONCh | 0.030 | 0.041 | 0.022 | 0.023 | 0.021 | 0.035 | 0.045 | 0.035 | 0.075 | 0.082 | 0.030 | 0.009 | 0.064 | 0.007 | 0.000 | 0.154 | 0.218 | 0.238 | 0.334 | 0.390 | 0.675 |
| HoJa | 0.061 | 0.063 | 0.041 | 0.046 | 0.047 | 0.051 | 0.063 | 0.052 | 0.103 | 0.109 | 0.037 | 0.037 | 0.092 | 0.028 | 0.016 | 0.000 | 0.190 | 0.350 | 0.370 | 0.385 | 0.718 |
| WaJa | 0.052 | 0.060 | 0.035 | 0.040 | 0.039 | 0.048 | 0.069 | 0.049 | 0.100 | 0.099 | 0.041 | 0.031 | 0.072 | 0.033 | 0.015 | 0.024 | 0.000 | 0.370 | 0.438 | 0.457 | 0.660 |
| RACh | 0.045 | 0.048 | 0.032 | 0.031 | 0.022 | 0.037 | 0.056 | 0.043 | 0.100 | 0.096 | 0.044 | 0.014 | 0.064 | 0.019 | 0.021 | 0.041 | 0.042 | 0.000 | 0.146 | 0.348 | 0.601 |
| BACh | 0.063 | 0.066 | 0.051 | 0.047 | 0.038 | 0.044 | 0.071 | 0.054 | 0.115 | 0.109 | 0.058 | 0.035 | 0.087 | 0.037 | 0.038 | 0.047 | 0.056 | 0.019 | 0.000 | 0.423 | 0.586 |
| ShoJa | 0.058 | 0.058 | 0.046 | 0.046 | 0.045 | 0.037 | 0.077 | 0.048 | 0.106 | 0.107 | 0.063 | 0.042 | 0.095 | 0.036 | 0.039 | 0.039 | 0.054 | 0.042 | 0.051 | 0.000 | 0.652 |
| JeJa | 0.077 | 0.079 | 0.065 | 0.061 | 0.058 | 0.057 | 0.090 | 0.068 | 0.125 | 0.117 | 0.068 | 0.059 | 0.111 | 0.067 | 0.070 | 0.091 | 0.082 | 0.064 | 0.065 | 0.077 | 0.000 |

**Table S3.** Genetic distance between pairs of populations estimated by F_ST_ (Above) and their p-values (Below) in five samples sites corresponding to three Sudan native cattle breeds.

|  | Baggara Daiwani | Baggara Nyakawi | Kenana | Butana Bu_Atbara | Butana Bu_Qadarif |
| --- | --- | --- | --- | --- | --- |
| Baggara Daiwani | 0 | 0.0002 | 0.0112 | 0.0078 | 0.0118 |
| Baggara Nyakawi | 0.450 | 0 | 0.0062 | 0.0097 | 0.0073 |
| Kenana | < 0.0001 | 0.009 | 0 | 0.0079 | 0.0107 |
| Butana Bu Atbara | 0.018 | < 0.0001 | 0.054 | 0 | 0.0086 |
| Butana Bu Qadarif | < 0.0001 | 0.027 | < 0.0001 | 0.063 | 0 |

**Table S4.** BoLa-DRB3 alleles present in Sudan native cattle breeds that were previously reported in African cattle.

| **BoLA-DRB3 Allele** | **Breeds** | **Reference** |
| --- | --- | --- |
| BoLA-DRB3*022:10 | Sudan Nyalawi | Salim B, unpublished^1^ |
| BoLA-DRB3*022:12 | Sudan Nyalawi | Salim B, unpublished^1^ |
| BoLA-DRB3*024:01 | African Bos indicus, Sudan Kenana, Ghana Sanga | Mikko and Andersson, *1995;* Acheampong D.A., Unpublished^1^; Salim B, unpublished^1^ |
| BoLA-DRB3*024:06 | Ghana Sanga | Acheampong D.A., Unpublished^1^ |
| BoLA-DRB3*032:03 | Sudan Kenana | Salim B, unpublished^1^ |
| BoLA-DRB3*100:01 | Baggara, Butana | Salim B, unpublished^1^ |
| BoLA-DRB3*107:01 | Butana | Salim B, unpublished^1^ |

^1^ IPD–MHC database direct submission.

**Table S5.** BoLA-DRB3 alleles shared between the Sudan breeds and Zebu, Southeast Asia and/or Creole Americas breed groups and absent in the European breeds.

| **BoLA-DRB3 Allele** | **Breeds** | **Reference** |
| --- | --- | --- |
| BoLA-DRB3*003:02:01 | Sudan, SE Asia, Zebu, N´Dama | Gelhaus *et al*., 1995 |
| BoLA-DRB3*005:02 | Sudan, SE Asia, Wagyu | Mikko and Andersson, *1995,* Takeshima *et al*., 2011 |
| BoLA-DRB3*020:03 | Sudan, SE Asia, Zebu, Brahman | Maillard *et al*., 1999 |
| BoLA-DRB3*023:01 | Sudan, SE Asia, Zebu, | Mikko and Andersson, *1995;* |
| BoLA-DRB3*025:01:01 | Sudan, SE Asia, Zebu | Mikko and Andersson, *1995* |
| BoLA-DRB3*027:04 | Sudan, SE Asia, Zebu, Boran | Gelhaus *et al*., 1995 |
| BoLA-DRB3*030:01 | Sudan, SE Asia, Zebu, Boran | Gelhaus *et al*., 1995 |
| BoLA-DRB3*039:01 | Sudan, SE Asia, Zebu, Ethiopian Arsi | Ballingall *et al*., 1996, Ballingall K.T.^1^ |

^1^ IPD–MHC database direct submission.

**Fig. S1** Maximum Parsimony tree constructed from the amino-acid residues located in the antigen-binding site (ABS) by all reported *BoLA-DRB3* alleles and the seven new ones (BoLA-DRB3*004:02Sp2, BoLA-DRB3*011:02Sp, BoLA-DRB3*018:01Sp, BoLA-DRB3*021:01sp, BoLA-DRB3*024:18Sp, BoLA-DRB3*027:05sp, and BoLA-DRB3*032:01sp). Numbers are bootstrap percentages that support each node. Bootstrapping was carried up with 1000 replicates to access the reliability of individual branches. Bag = Baggara, But = Butana, Ken = Kenana. Arrows indicate novel alleles.

**
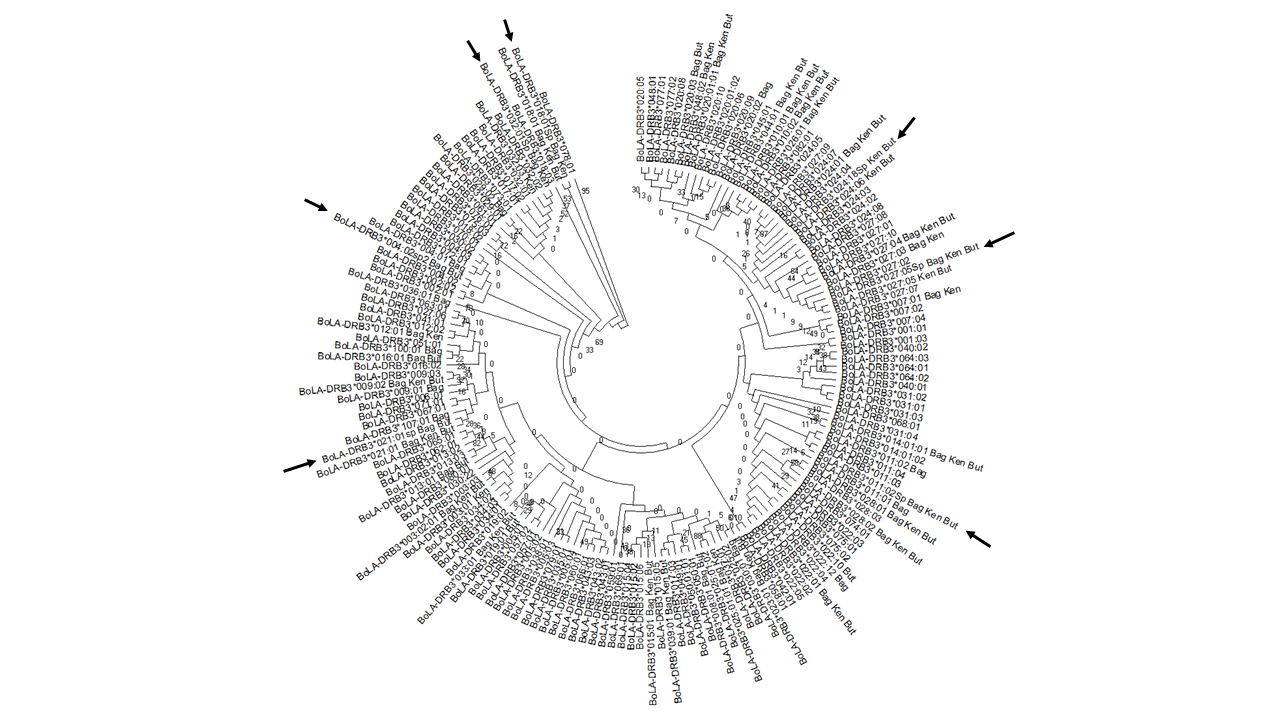
**

**Fig. S2** Cumulative gene frequency plot of BoLA-DRB3 alleles in Baggara (blue), Butana (violet) and Kenana (orange) cattle populations from Sudan.

**Figure S3a-e.** Principal component analysis of BoLa-DRB3 gene pocket amino acid motifs frequencies in 22 Breeds/populations: a. Pocket 1, b. Pocket 4, c. Pocket 6, d. Pocket 7, and e. Pocket 9. Bag = Baggara, But = Butana, Ken = Kenana, BW = Pyer Sein, GR = Shwe Ni, NaPh = Philippine Native, BrPh = Philippine Brahman, NeBo = Bolivian Nellore, GirBo = Bolivian Gir, and BrxNe = Peruvian Brahman × Nellore crossbreed, WaJa = Japanese Black, HoJa = Holstein, ShJa = Japanese Shorthorn, JeJa = Japanese Jersey, HeCh = Chilean Hereford, BACh = Chilean Black Angus, RACh = Chilean Red Angus, ONCh = Chilean Overo Negro, OCCh = Chilean Overo Colorado, CrHV = Hartón del Valle Creole, CrYa = Yacumeño Creole, and CrAl = Highland Creole.

a.

**
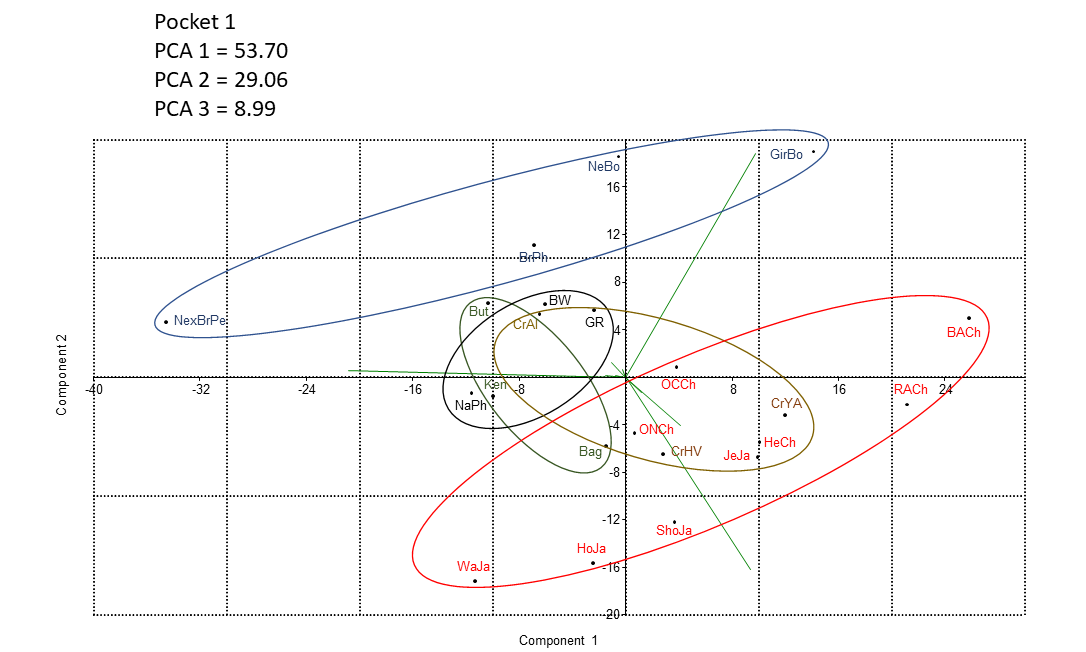
**

b.

**
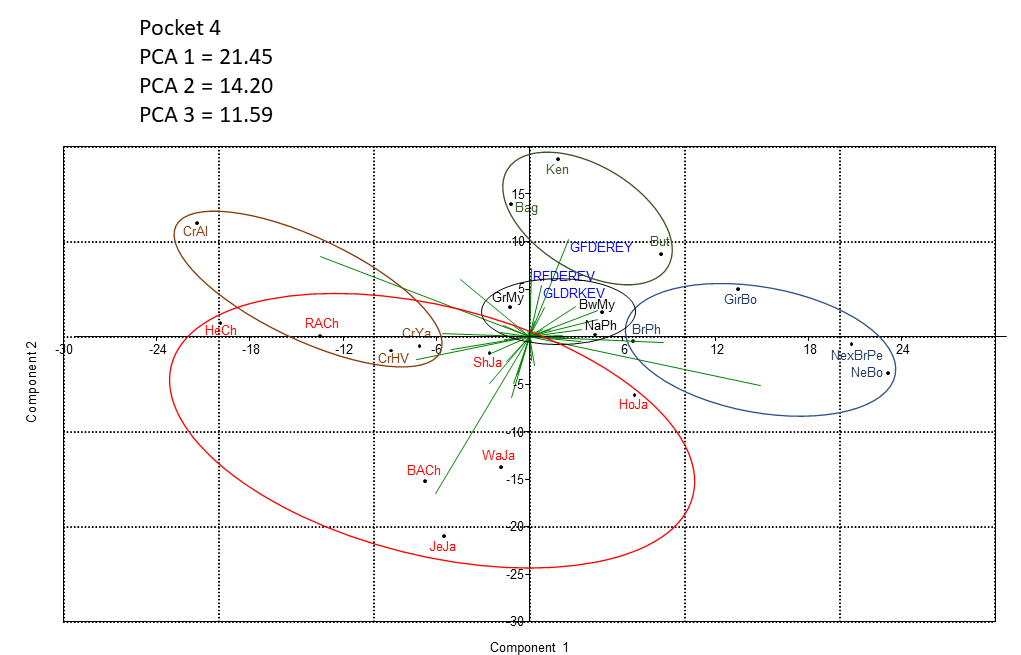
**

**c.**

**
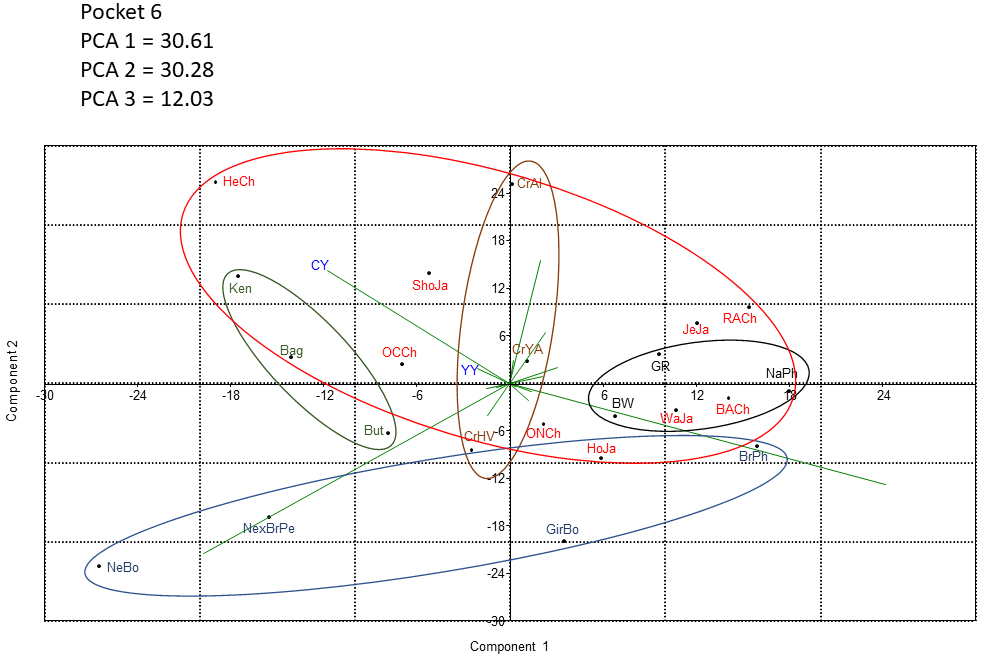
**

**d.**

**
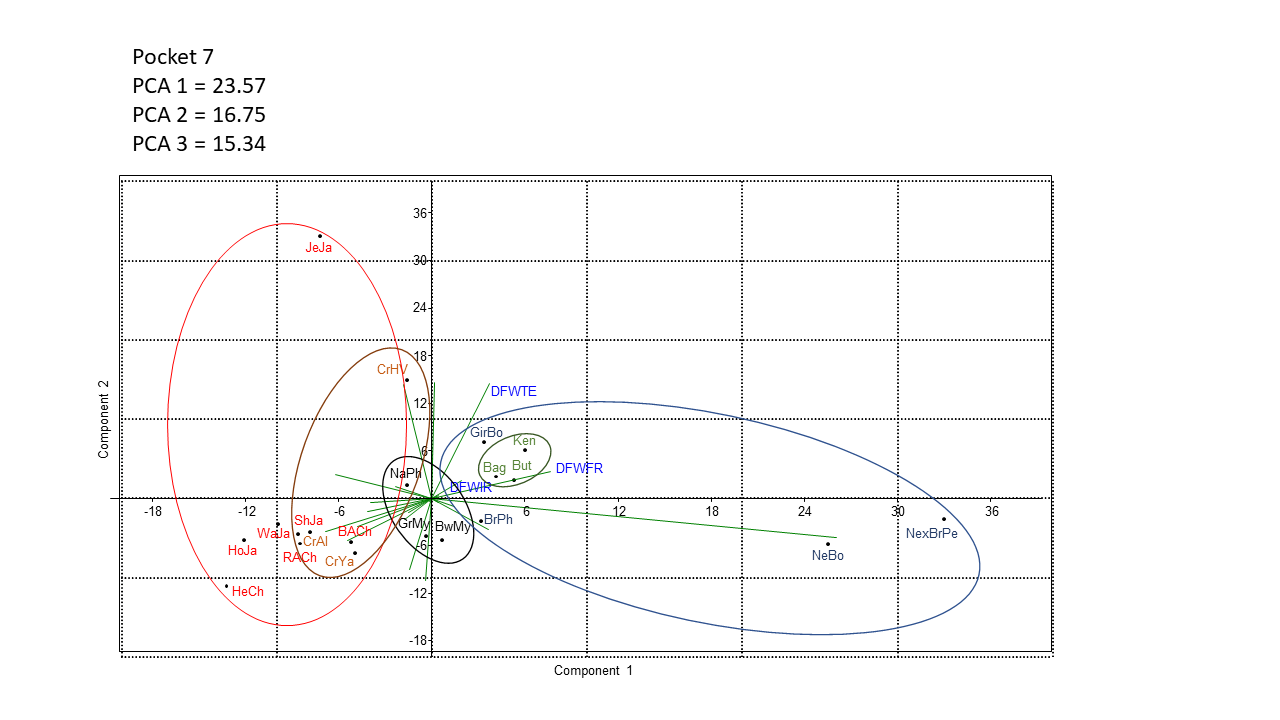
**

**e.**

**
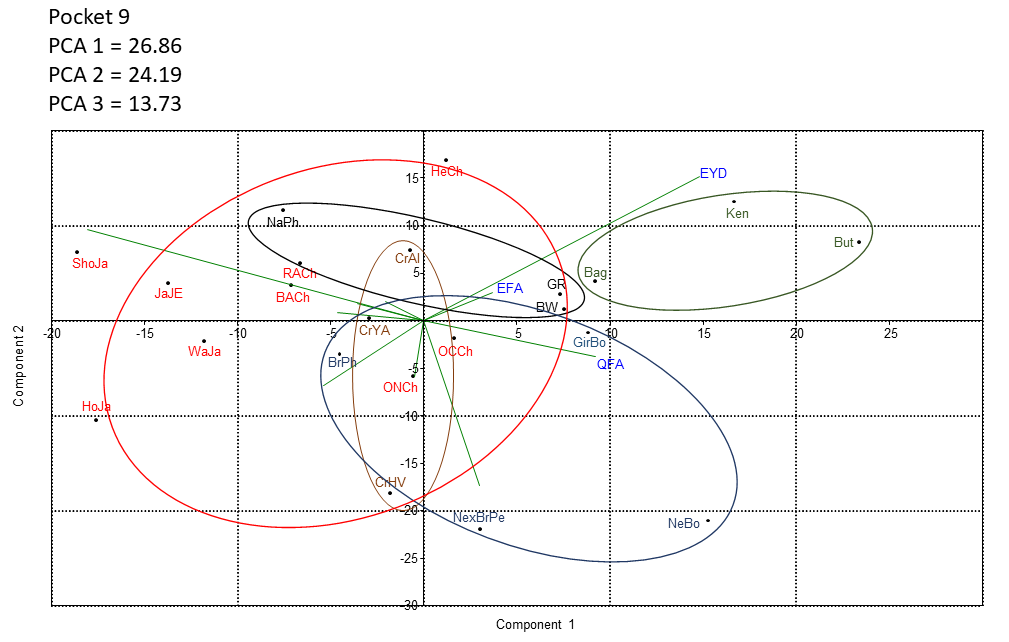
**

**Figure S4.** Geographic location of the sampling sites (indicated with asterisk) of Baggara, Butana and Kenana cattle populations form Sudan.


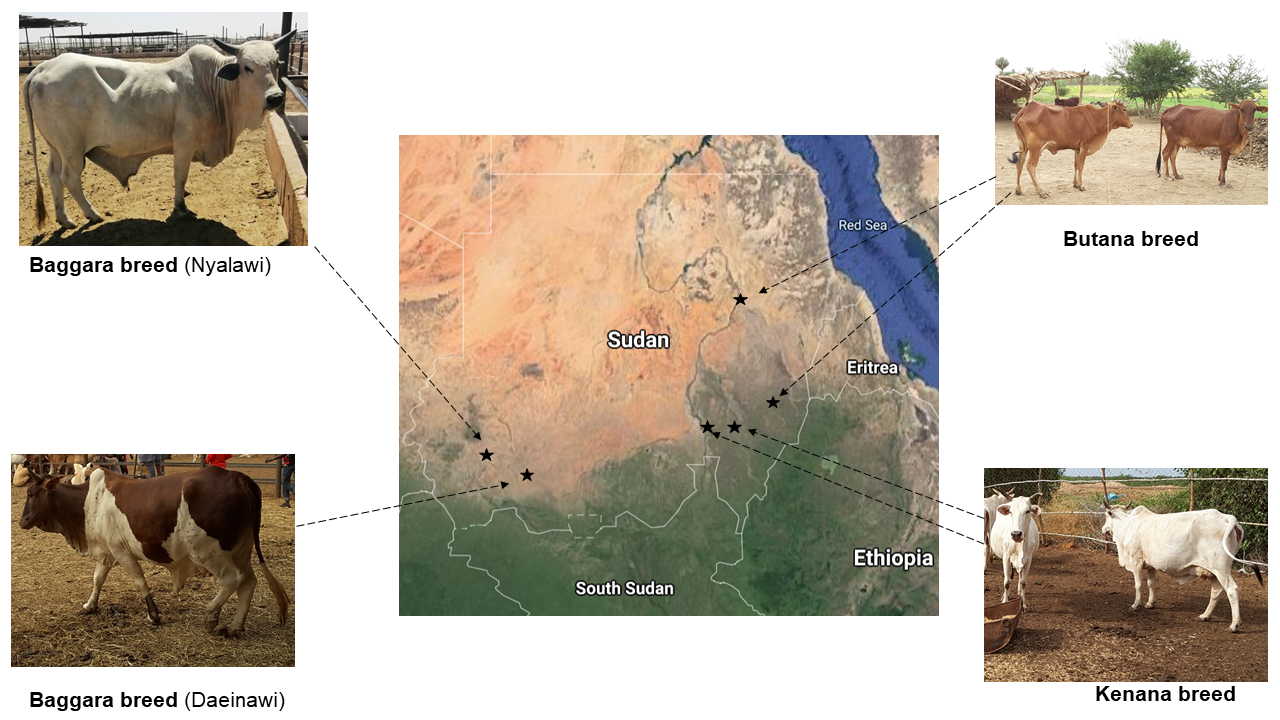

Supplement: Supplementary file 1 — Supplementary Information. [file 41598_2021_96330_MOESM1_ESM.docx]
